# Clergymen and Physicians

**Published:** 1856-06

**Authors:** 


### CLERGYMEN AND PHYSICIANS.

The medical public has been entertained recently with a discussion as to the relation subsisting between the members of these professions and the obligations of the latter to the former. Time out of mind it has been the custom for the medical man to give his professional services gratuitously to the ministers of the gospel and their families. But it would appear that the justice of such a proceeding is doubted in certain quarters and we confess that in certain cases such gratuitous labor, care, and responsibility is without the semblance of justice.

When a clergyman is found in the possession of wealth, which is not often the case, then we think he is not entitled to the exemption, particularly when he persists in exacting payment for *his* services to his church and over his flock. When, too, a physician is poor and the minister in moderate circumstances, we think that, in that case, the divine, if he be a conscientious man, will recognise the principle of *lex justitia* and act accordingly. And, again, in all instances when the divine has made use of his influence in favor the of various *isms*, he should then, if the services of the regular medical man have have been required and given, such divine should be required to pay to the uttermost farthing. He has been engaged in undervaluing the capacities of true medical science, and his influence has been potent against it, and if after this he shall be the recipient of its benefits, in any manner, he should be made to feel that he has been fortunate beyond his deserts, even in remunerating the hand that he has been attempting to paralyze in its efforts for good.

Then, again, a difficulty will arise, as it occurs to us, in determining who are really ministers of the gospel, If he be a Catholic physician, he must look upon protestant ministers as heretics, and ought not to claim an exemption, even if he extended it to the ministers of his own church. Then, again, we have Mormon priests who may claim the benefits, and the physician may very justly hold that he is engaged in the dissemination of doctrines the very antipodes to those taught in the Bible.

We know no way in which to settle these difficulties, than to leave it to the discretion of the physician himself. Clergymen in the main are poorly supported, particularly in the west, and we therefore have no disposition to change a custom, which for many long years we have observed, and still less are we disposed to require payment, so long as they do not despise the teachings contained in their own great "text-book," and continue to use their influence in favor of legitimate medicine.

